# Long-term effectiveness and safety of varenicline and nicotine replacement therapy in people with neurodevelopmental disorders: A prospective cohort study

**DOI:** 10.1038/s41598-019-54727-5

**Published:** 2019-12-20

**Authors:** Taha Itani, Dheeraj Rai, Tim Jones, Gemma M. J. Taylor, Kyla H. Thomas, Richard M. Martin, Marcus R. Munafò, Neil M. Davies, Amy E. Taylor

**Affiliations:** 1 Medical Research Council Integrative Epidemiology Unit at the University of Bristol, Bristol, BS8 2BN United Kingdom; 2 School of Psychological Science, 12a Priory Road, Bristol, BS8 1TU United Kingdom; 3 Centre for Academic Mental Health, Bristol Medical School, University of Bristol, Barley House, Oakfield Grove, Bristol, BS8 2BN United Kingdom; 4 Avon & Wiltshire Partnership NHS Mental Health Trust, Bristol, UK; 5 NIHR Biomedical Research Centre at the University Hospitals Bristol NHS Foundation Trust and the University of Bristol, Bristol, United Kingdom; 6 The National Institute for Health Research Applied Research Collaboration West (NIHR ARC West) at University Hospitals Bristol NHS Foundation Trust, Bristol, United Kingdom; 7 Addiction and Mental Health Group (AIM) Department of Psychology, University of Bath, Claverton Down, Bath, BA2 7AY United Kingdom; 8 Population Health Sciences, Bristol Medical School, University of Bristol, Bristol, BS8 2BN United Kingdom

**Keywords:** Psychology, Medical research

## Abstract

This study aimed to determine the effectiveness and safety of varenicline versus NRT for smoking cessation in people with neurodevelopmental disorders, compared to those without, at up to four years after exposure. We analysed electronic medical records from the Clinical Practice Research Datalink using three different statistical approaches: multivariable logistic regression, propensity score matching (PSM), and instrumental variable analysis. Exposure was prescription of varenicline versus NRT and the primary outcome was smoking cessation at 2-years. We included 235,314 people aged 18 and above with eligible smoking cessation prescriptions in the effectiveness analysis. Smokers with neurodevelopmental disorders were 48% less likely (95% confidence interval: 42%, 54%) to be prescribed varenicline than NRT, compared to smokers without neurodevelopmental disorders. At 2-year follow-up, smokers with neurodevelopmental disorders prescribed varenicline were 38% more likely to quit smoking (95% confidence interval: 6%, 78%). Similar results were obtained using PSM and instrumental variable analyses. There was little evidence showing that varenicline increased the likelihood of mental health related adverse events in people with neurodevelopmental disorders. Varenicline is less likely to be prescribed to people with neurodevelopmental disorders despite results suggesting it is more effective than NRT and little evidence of increased likelihood of mental health related adverse events.

## Introduction

Intellectual disability, autism spectrum disorders (ASD) and attention deficit hyperactivity disorder (ADHD) are neurodevelopmental disorders with a cumulative prevalence of at least 5% in the general population. There is increasing evidence that people with neurodevelopmental disorders experience health inequalities and up to 16 years of premature mortality as compared to those without these conditions^1^. For instance, those with intellectual disabilities are more likely to have worse physical and mental health and receive poorer quality health care when compared to the general population in the United States^2^, Canada^3^, and the UK^4,5^.

Smoking prevalence amongst people with ADHD is higher than that in the general population^6^, individuals with ADHD start smoking at an earlier age^6,7^ and find it more difficult to quit smoking compared to the general population^8^. Studies conducted in developed nations estimate that 36 to 42% of adults with ADHD smoke compared to 26% in those without ADHD^6,9^. These prevalence estimates were based on old data (prior to 1997) and may not reflect the current trends. Conversely, there is evidence to suggest that smoking prevalence is low amongst people with ASD. A Swedish study of 95 adults with ASD found that 13% were smokers^10^ compared to 19% in the general population^11^. Variations in smoking prevalence might be due to various reasons including different neurobiological underpinnings (e.g. the role of dopaminergic pathways in ADHD), phenotypic differences (e.g. impulsivity and high-risk behaviours in ADHD vs typically more rule abiding behaviours in autism) or access to smoking due to disorder severity, as individuals with mild to moderate intellectual disabilities have higher smoking rates than those with more severe intellectual disabilities^12^. However, there is limited evidence on smoking rates amongst people with neurodevelopmental disorders in the UK: amongst 435 adults with learning disabilities in one large urban area of central UK a smoking prevalence of 6.2% was observed (lower than the general population)^13^.

There is a lack of evidence about the effectiveness of smoking cessation medications amongst smokers with neurodevelopmental disorders. Varenicline, a nicotinic receptor partial agonist, is a more effective smoking cessation agent than placebo, NRT and bupropion in randomised controlled trials^14,15^ and in the general population^16^. However, no studies have been conducted to assess the effectiveness of varenicline on smoking cessation amongst people with neurodevelopmental disorders.

Since its launch, spontaneous reporting systems have highlighted concerns about the safety of varenicline, particularly in relation to mental health^17^. These concerns prompted the Medicines and Healthcare Products Regulatory Agency (MHRA) to issue safety warnings about varenicline in the UK^18^ and the U.S. Food and Drug Administration (FDA) to require the addition of a black box warning to the labelling of varenicline (removed in 2016)^19^. Empirical studies indicate no clear evidence of an increased risk of neuropsychiatric side effects including suicidal behaviour in the general and psychiatric populations associated with varenicline (versus NRT or placebo)^17,20–22^. However, these safety issues have not been investigated in people with neurodevelopmental disorders.

While considering that people with neurodevelopmental disorders may suffer from health inequalities and that there is a scarcity of information about smoking prevalence and smoking cessation treatment, shedding more light on these potentially avoidable issues could foster health equality in this patient group. Hence, we aimed to: (1) describe smoking prevalence and rates of prescribing of smoking cessation medication in people with neurodevelopmental disorders in primary care from 2004 to 2015, as compared to those without these disorders, and (2) determine the effectiveness and safety of varenicline versus NRT for smoking cessation in people with neurodevelopmental disorders, compared to those without these disorders, at 3, 6, 9 months and 1, 2, 4 years after exposure.

## Results

### Population characteristics

Between September 1^st^, 2006 and January 25^th^, 2016, there were 235,314 people with eligible prescriptions for smoking cessation medications (Please see eFigure 1 in the online supplemental data displaying the number of patients excluded and reasons for exclusion.). Amongst those with neurodevelopmental disorders (N = 2,346), 1,882 and 464 smokers were prescribed NRT and varenicline, respectively. People with neurodevelopmental disorders were on average about 10 years younger, more likely to be males, more likely to have alcohol and drug misuse, and were diagnosed with more lifetime mental health disorders than those without neurodevelopmental disorders (Table 1).

**Table 1:** Baseline characteristics of patients with or without neurodevelopmental disorders by exposure group, N (%).

Characteristic	Any neurodevelopmental disorder (N = 2,346)			No neurodevelopmental disorder (N = 232,968)		
	NRT (N = 1,882)	Varenicline (N = 464)	Total	NRT (N = 157,854)	Varenicline (N = 75,114)	Total
Age at time of first prescription^a^	35.5 (14.4)	34.1 (13.2)	35.3 (14.2)	46.4 (15.2)	44.5 (13.2)	45.8 (14.8)
Sex (male)	1,194 (63.4%)	287 (61.9%)	1,481 (63.1%)	72,666 (46.0%)	37,408 (49.8%)	110,074 (47.3%)
Multiple deprivation score (IMD)^b†^	4	4	4	3	3	3
Number of GP visits 1-year prior to first prescription^a^	9.4 (8.9)	7.8 (9.7)	9.1 (9.1)	7.9 (7.4)	6.3 (6.1)	7.4 (7.0)
BMI^a†^	27.1 (6.7)	26.2 (6.2)	26.9 (6.6)	26.4 (5.7)	26.5 (5.4)	26.5 (5.6)
Year of first prescription^b^	2010	2011	2010	2009	2010	2009
Days of history^a^	2,733.3 (1871.0)	3,011.7 (2135.1)	2,788.4 (1928.8)	3,058.1 (1908.1)	3,165.8 (1985.4)	3,092.8 (1934.0)
Comorbidity ever (Charlson Index)	727 (38.6%)	185 (39.9%)	912 (38.9%)	59,116 (37.5%)	23,860 (31.8%)	82,976 (35.6%)
Alcohol misuse ever	197 (10.5%)	54 (11.6%)	251 (10.7%)	13,797 (8.7%)	4,716 (6.3%)	18,513 (8.0%)
Drug misuse ever	109 (5.8%)	24 (5.2%)	133 (5.7%)	4,866 (3.1%)	1,432 (1.9%)	6,298 (2.7%)
Self-harm ever	415 (22.1%)	79 (17.0%)	494 (21.1%)	16,884 (10.7%)	6,582 (8.8%)	23,466 (10.1%)
Ever anxiety and stress related disorders	626 (33.3%)	125 (26.9%)	751 (32.0%)	43,906 (27.8%)	17,268 (23.0%)	61,174 (26.3%)
Other behavioural/neurologic disorder ever	346 (18.4%)	66 (14.2%)	412 (17.6%)	7,322 (4.6%)	2,529 (3.4%)	9,851 (4.2%)
Ever depression	851 (45.2%)	187 (40.3%)	1,038 (44.3%)	64,709 (41.0%)	25,936 (34.5%)	90,645 (38.9%)
Ever schizophrenia and non-affective psychoses	233 (12.4%)	10 (2.2%)	243 (10.4%)	4,030 (2.6%)	431 (0.6%)	4,461 (1.9%)
Ever antidepressants	1,049 (55.7%)	214 (46.1%)	1,263 (53.8%)	78.808 (50.0%)	32,044 (42.7%)	110,852 (47.6%)
Ever antipsychotics	632 (33.6%)	83 (17.9%)	715 (30.5%)	28,515 (18.1%)	9,722 (12.9%)	38,237 (16.4%)
Ever hypnotics/anxiolytics	821 (43.6%)	169 (36.4%)	990 (42.2%)	59,509 (37.7%)	24,985 (33.3%)	84,494 (36.3%)
Ever mood stabilisers	278 (14.8%)	21 (4.5%)	299 (12.8%)	7,365 (4.7%)	1,783 (2.4%)	9,148 (3.9%)

^a^Data presented are mean and standard deviation. ^b^Data presented are median. ^†^Missing data: BMI data was missing for 14.1% (N = 33,059); IMD data was missing for 43.6% (N = 102,657).

### Prevalence of smoking and of prescribing of smoking cessation medication over time

**Figure 1:**
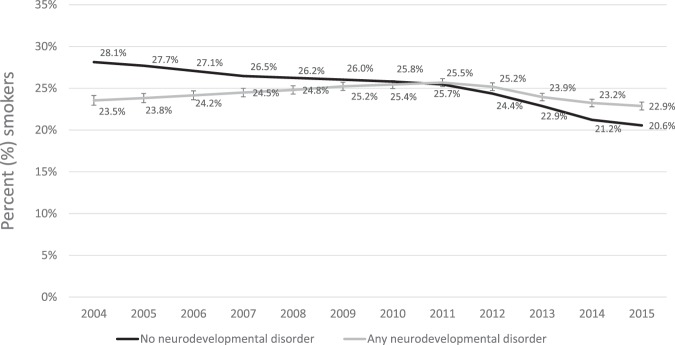
Age and sex standardised percentage (%) of primary care patients with an electronic medical record indicating smoking, from 2004 to 2015, in people with or without neurodevelopmental disorders.

The age and sex standardised prevalence of smoking amongst people with neurodevelopmental disorders was 23.5% in 2004, rising to 25.7% in 2010 and decreasing to 22.9% by 2015 (Fig. 1). People with ADHD had the highest standardised smoking prevalence, ranging between 34.7% and 37.7% in the study period while people with ASD had the lowest standardised smoking prevalence, ranging between 14.9% and 15.7% in the study period. The age and sex standardised smoking prevalence amongst people with intellectual disabilities ranged from 21.8% in 2004 to 20.1% in 2015 (eFigure 2). Amongst those with no neurodevelopmental disorders, smoking rates decreased steadily from 28.1% in 2004 to 20.6% in 2015 (Fig. 1).

**Figure 2:**
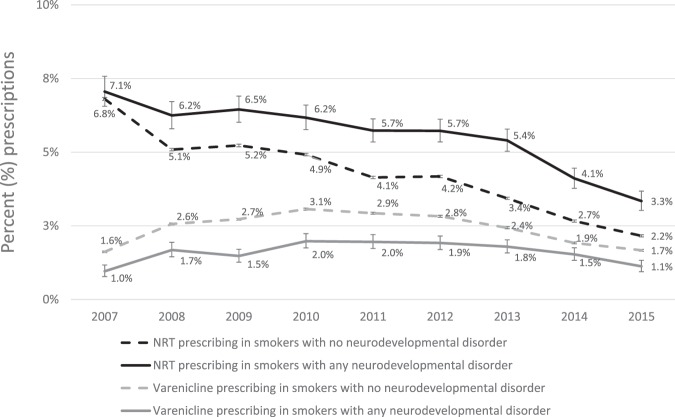
Age and sex standardised prescription prevalence of varenicline or NRT in primary care, from 2007 to 2015, in smokers with any neurodevelopmental disorder, compared to smokers with no neurodevelopmental disorder.

Standardised NRT prescribing rates fell from 7% to 2–3% between 2007 and 2015 in smokers with and without neurodevelopmental disorders. Varenicline prescribing increased between 2007 and 2011 and then fell between 2011 and 2015 in smokers with and without neurodevelopmental disorders. From 2007 to 2015, NRT prescribing was higher amongst smokers with neurodevelopmental disorders, while varenicline prescribing was higher amongst smokers without neurodevelopmental disorders (Fig. 2).

### The effectiveness of varenicline versus NRT for smoking cessation

**Figure 3:**
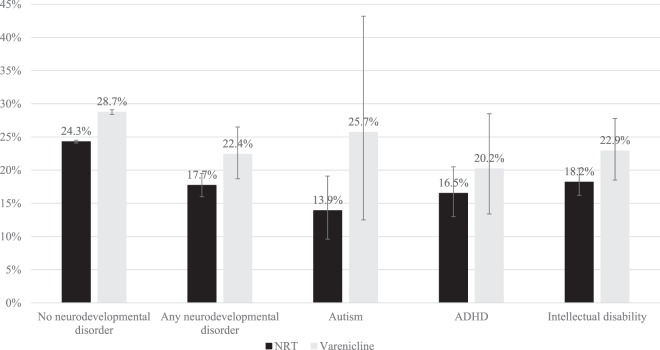
Percentage (%) of patients with an electronic medical record indicating smoking cessation at 2-years follow-up, by exposure, and neurodevelopmental disorder.

Smokers with neurodevelopmental disorders were 48% (95% confidence interval: 42% to 54%) less likely to be prescribed varenicline than NRT, compared to smokers without neurodevelopmental disorders (eTable 3). Amongst smokers with and without neurodevelopmental disorder, varenicline was associated with higher quit rates than NRT at all follow-up time points (Fig. 3, Table 2 and eTable 4, for partially adjusted models see eTable 5).

**Table 2:** Stratified by mental disorder: Fully adjusted odds ratios and 95% confidence intervals for the association between prescription of varenicline versus NRT and smoking cessation at 3, 6 and 9-months and 1, 2, and 4-years after prescription.

Fully adjusted odds ratio (95% confidence interval)^†^						
	3-months	6-months	9-months	1-year	2-years	4-years
No neurodevelopmental disorder (N = 232,968)	1.42 (1.38 to 1.47)	1.45 (1.41 to 1.50)	1.39 (1.36 to 1.43)	1.33 (1.30 to 1.37)	1.25 (1.22 to 1.27)	1.17 (1.14 to 1.19)
Any neurodevelopmental disorder (N = 2,344)	1.45 (1.03 to 2.05)	1.63 (1.23 to 2.17)	1.61 (1.23 to 2.12)	1.31 (0.99 to 1.73)	1.38 (1.06 to 1.78)	1.32 (1.02 to 1.71)
Autism (N = 258)	5.13 (1.24 to 21.25)	9.27 (1.91 to 45.01)	5.87 (1.70 to 20.27)	4.87 (1.52 to 15.60)	3.16 (1.09 to 9.20)	1.96 (0.74 to 5.19)
ADHD (N = 524)	1.40 (0.63 to 3.13)	1.50 (0.74 to 3.03)	1.81 (0.99 to 3.33)	0.91 (0.50 to 1.66)	1.08 (0.62 to 1.91)	1.13 (0.66 to 1.95)
Intellectual disability (N = 1,720)	1.39 (0.92 to 2.12)	1.62 (1.16 to 2.27)	1.55 (1.11 to 2.15)	1.43 (1.03 to 1.97)	1.40 (1.03 to 1.91)	1.33 (0.97 to 1.83)

^†^Fully adjusted models were adjusted for: age, sex, days in history, IMD, number of GP visits 1-year prior to first prescription, BMI, year of first prescription, history of major physical morbidity (Charlson Index), alcohol misuse ever, drug misuse ever, depression ever, neurotic disorder ever, schizophrenia ever, self-harm ever, antidepressant prescription ever, antipsychotic prescription ever, hypnotics/anxiolytics prescription ever, other psychotropic medication ever, and other behavioral/neurologic disorder ever. Missing BMI and IMD values were imputed using multiple imputation.

After adjusting for all covariates, smokers with no neurodevelopmental disorder prescribed varenicline were 25% (95% confidence interval: 22% to 27%) more likely to abstain from smoking after two years of follow-up. At 2-years follow-up, smokers with neurodevelopmental disorders prescribed varenicline were 38% (95% confidence interval: 6% to 78%) more likely to quit smoking in the fully-adjusted model. Estimates were similar across the time points for the group with neurodevelopmental disorders but were imprecise due to smaller sample size (Table 2).

The overall trend in the propensity score matched models was consistent with the estimates produced by the multivariable logistic regression models (eTable 6). The instrumental variable models indicated that smokers with and without any neurodevelopmental disorder were more likely to quit smoking after 2-years when prescribed varenicline vs NRT; the risk difference per 100 people treated (this corresponds to the number of people out of 100 who would be expected to quit smoking when prescribed varenicline vs. NRT) was 11.98 (0.29 to 23.67) and 5.31 (3.67 to 6.95), respectively (eTable 7).

### Safety of varenicline versus NRT

Amongst patients with neurodevelopmental disorders, there were seven cases of death by suicide in those who received NRT and no cases in the varenicline group.

There was little evidence that varenicline increased the likelihood of any of the mental health related adverse events (eTables 8–11).

## Discussion

### Principal findings

To our knowledge, this is the largest study to estimate smoking prevalence amongst patients with neurodevelopmental disorders using nationally-representative primary care data and the first to assess the effectiveness and safety of varenicline for smoking cessation in this patient group. Our findings indicate that smoking prevalence is higher in people with neurodevelopmental disorders (with the exception of patients with ASD) than people without these disorders. These individuals are less likely to be prescribed varenicline than NRT. However, there was little evidence that the effectiveness of varenicline over NRT is different in this group compared to the general population. The results were comparable across the three analytical methods, although instrumental variable estimates were imprecise. Furthermore, our analysis of safety outcomes in patients with neurodevelopmental disorders found little evidence that varenicline was associated with increased likelihood of suicide, self-harm, depression or anxiety. These results were similar across all three analytical methods, albeit with limited power to produce precise estimates on varenicline safety.

### Strengths and limitations

The main strengths of this study include the use of a representative population-based primary care data and triangulating three different analytical methods^23^ to address confounding^24^. The CPRD is representative of the UK primary care population^25^, thus our results are likely to be generalizable to the UK and similar countries. The large sample size allowed us to investigate smoking prevalence and cessation in patients with relatively rare neurodevelopmental disorders. Additionally, we used multiple imputation to handle missing data on BMI and IMD^26^ to reduce selection-bias. The consistent results across our different statistical modeling techniques suggest that these are less likely to be driven by residual confounding.

A limitation is that we had no information on compliance with taking prescribed smoking cessation medications as intended. Therefore, the efficacy of varenicline might be underestimated if smokers were less likely to adhere to their treatment regimen. Our results might not be generalizable to all people with intellectual disabilities since it is considered that the ‘hidden majority’^27^ of those with mild forms of the disorder tend to have less access to screening interventions and might be underdiagnosed in the CPRD. Moreover, smoking records may not reflect the up-to-date smoking status which might over- or underestimate the smoking prevalence. Selection bias could be a concern since the CPRD database only includes patients who seek primary care services. Additionally, data on other important confounders such as parent and peer relations are not captured by the CPRD. Finally, our study lacked statistical power to detect small associations between the exposure and the outcome.

### Comparison with other studies

There is a lack of population-based studies estimating the smoking prevalence in people with neurodevelopmental disorders. We observed a higher smoking prevalence 37.9% (95% CI: 36.3% to 39.5%) amongst ADHD patients in 2010 than previously reported in the THIN database 27.2% (95% CI: 21.0% to 33.3%) in 2009–2010^28^. This may be due to the difference in denominators (i.e., people with missing smoking records were excluded from the denominator). We observed higher smoking rates in people with neurodevelopmental disorders as compared to those without. This was consistent with a study in people with and without longstanding mental health disorders^29^. Meanwhile, our data indicate a steady decline of smoking rates in people with no neurodevelopmental disorders during the study period which is concordant with smoking estimates in the UK’s general population^30^.

This study indicates a declining trend in NRT prescriptions for smokers with and without developmental disorders. This decline was mirrored in a study that used the THIN data^31^. Regarding varenicline, our data show an initial increase in its prescriptions to smokers in both groups between 2008 and 2011. After varenicline entered the drug market in the UK in late 2006, it became the second most prescribed drug for smoking cessation in the UK^31^. From 2007 to 2015, varenicline prescribing was higher amongst smokers without neurodevelopmental disorders which may be indicative of increased cautiousness by physicians to prescribe varenicline to people with neurodevelopmental disorders. The latter could be related to the safety warnings that were raised about varenicline by the MHRA in the UK^18^. However, it is worth noting that these warnings have been dropped since 2016 based on the results from the EAGLES clinical trial^21^. The steady decline in NRT prescriptions and the eventual decline in varenicline prescriptions after 2011 may be partially attributed to the rise in e-cigarette popularity^32^, to the availability of over-the-counter smoking cessation medications, or indicate a decline in provision of smoking cessation services.

Our safety analysis of varenicline use for smoking cessation in patients with neurodevelopmental disorders as compared to NRT revealed little evidence of increased likelihood of adverse events such as self-harm, depression, anxiety and prescriptions of antidepressants and hypnotics/anxiolytics. These results were consistent with evidence from observational^20,33^ and clinical studies^16,21,22^. These mounting and consistent findings that find little evidence that varenicline has few detectable mental health side effects compared to NRT should provide reassurance to clinicians.

People with neurodevelopmental disorders should have access to the most effective smoking cessation treatments to reduce the burden of smoking-related health inequalities in this group. Although the NHS introduced a Directed Enhanced Service (DES) in 2009^34^ to provide annual health checks for people with learning disabilities (those were the largest group in our cohort of people with neurodevelopmental disorders) and ultimately improve their health, this has not translated to better smoking cessation advocacy in the primary care setting. Our results signal that such efforts should be refined to tackle the high prevalence of smoking and low rate of prescribing varenicline for smoking cessation in patients with neurodevelopmental disorders.

We found that smoking prevalence was higher in people with neurodevelopmental disorders (with the exception of ASD) as compared to those without these disorders. Varenicline was less likely to be prescribed to patients with neurodevelopmental disorders although our data suggests that it is more effective than NRT in achieving smoking abstinence in this patient group. We found little evidence that varenicline produced higher adverse events in terms of suicidal behaviour, depression, and anxiety than NRT in patients with neurodevelopmental disorders. This evidence may be useful in refining clinical guidelines on the use of varenicline for smoking cessation and reducing health inequalities in people with neurodevelopmental disorders.

## Methods

### Study design and data source

We conducted a prospective cohort study using the UK Clinical Practice Research Datalink (CPRD). The CPRD comprises anonymised data from over 13 million primary care patients gathered by 683 general practices (GPs) across the UK since 1987. Data include, but are not limited to: demographics, laboratory tests, imaging, diagnoses, therapies, hospital referrals and health-related behaviours^35^. The CPRD is broadly representative of the UK general population regarding key demographics such as age, sex, and ethnicity^35^. To ensure the integrity of the data, the CPRD data are validated, audited and quality checked^36^.

### Study population

We included people with Read codes indicating one of the following neurodevelopmental disorders (these were considered life-long conditions) at any time in the CPRD: (1) ASD (F84.0–84.1, F84.4–84.9), (2) intellectual disability (F70–79), and (3) ADHD (F90) and/or prescription of central nervous system stimulants (BNF chapter 4.4). There are no other licensed or off-licence indications for stimulant use other than ADHD. It is therefore common for epidemiological studies to use ADHD medications as a proxy for ADHD diagnoses^37^. People without the above Read codes were considered to have no neurodevelopmental disorder. Only people whose records originated from GPs that were up to standard and were deemed acceptable by the CPRD were included in the analysis (i.e., the included population had no gaps in their electronic medical records and had no missing key information such as year of birth, registration date or sex).

### Code lists

We extracted the variables for this study using medical and product codes within the CPRD. For a subset of the data, we used linked Office for National Statistics (ONS) mortality data to extract codes related to deaths by suicide. Where available, previously published lists were used^27^. Otherwise, code lists were created by consulting with field experts (RMM, DR, KHT) and by using the British National Formulary (BNF) and the International Classification of Diseases (ICD-10).

### Smoking status

We extracted smoking records from clinical files and additional data tables using Read codes specific to smoking, in addition to codes indicating prescription of smoking cessation medication extracted from therapy data files. Those were captured using prescribed medicines in BNF category 4.10.2 (Nicotine Dependence). According to the recent Quality and Outcomes Framework (QOF) guidelines (these are quality standards against which the priority areas for quality improvement in health and social care are set out), smoking status (non-smoker, current smoker, or ex-smoker) should be recorded by GPs annually for smokers, annually for ex-smokers for three consecutive years, and annually for non-smokers until they reach the age of 25^36^. These smoking records provide data that are consistent with smoking prevalence reported in representative population surveys^38^.

### Effectiveness and safety of varenicline vs NRT

#### Exposure measures

Individuals aged 18 years and older prescribed varenicline from 1^st^ September 2006 were compared to users of NRT (i.e., nicotine patches, gum, lozenges, mini-tabs and inhalers, defined using medicines in British National Formulary (BNF) category 4.10.2 as described above). Only first-time users of smoking cessation medications were included. First-time use of the smoking cessation therapies was defined as having no prior record of use of a related product in the preceding 18-months. We did not model treatment switching, as this is strongly related to patient characteristics. To ensure comprehensive assessment of baseline data and possible confounders, people who registered with the GP practice within 365 days of their first recorded smoking medication were excluded. We also excluded people who were initially prescribed NRT and varenicline simultaneously and those who were prescribed bupropion in the year before their first prescription of varenicline or NRT.

#### Outcome measures

The pre-specified primary outcome was smoking cessation at 2-years follow-up^39^. Smoking cessation was also assessed at 3, 6, and 9-months, and at 1 and 4-years after first prescription. Smoking status was ascertained by using each person’s most recent smoking record identified between cohort entry and each follow-up period (e.g., 3-months, 6-months, 1-year). The closest smoking record to each follow-up period was selected.

Safety-related outcomes were incident self-harm, depression, anxiety, and prescription of antidepressants and hypnotics/anxiolytics (see Supplementary Appendix), assessed at 3, 6, and 9-months, and at 1, 2 and 4-years after the index prescription of smoking cessation medications

#### Covariates

Covariates defined at the time of prescription included age, sex, index of multiple deprivation (IMD) that reflects the individual’s socioeconomic position, number of GP visits 1-year prior to first prescription, body mass index (BMI), year of first prescription, days registered in the CPRD, Charlson Index that is a measure of comorbidity, ever alcohol misuse, ever drug misuse, ever self-harm, history of mental health disorder (anxiety and stress related disorders, depression, bipolar disorder, schizophrenia and non-affective psychoses, eating and personality disorders), and history of psychotropic medication prescriptions (antidepressants, antipsychotics, hypnotics/anxiolytics, or mood stabilisers).

### Statistical analysis

#### Smoking prevalence

To estimate the prevalence of smoking in the target population, we divided the number of people with neurodevelopmental disorders aged 18 years and over who had Read codes indicating current smoking for each year between 2004 and 2015 by the total number of people with a smoking status code (indicating current or non/ex-smoking) each year between 2004 and 2015 (eTable 1). For comparison, smoking prevalence was also estimated amongst individuals without neurodevelopmental disorders.

#### Prescribing prevalence

The prevalence of varenicline and NRT prescribing amongst current smokers was calculated by dividing the number of prescriptions each year from 2007 to 2015 (there were no varenicline prescriptions for patients with neurodevelopmental disorders in 2006) by the number of current smokers in each year. Prevalence was estimated for people with and without neurodevelopmental disorders.

Smoking and prescribing prevalence rates were directly age- and sex- standardised to account for differences in age and sex between groups with and without neurodevelopmental disorders. We used the CPRD population in 2015 as our standard population for calculating standardised smoking rates, and the CPRD smoker population in 2015 as our standard population for calculating standardised prescribing prevalence rates. Age was grouped into five categories (18–24, 25–34, 35–49, 50–59, 60+ years).

#### Varenicline effectiveness and safety

The effectiveness and safety of varenicline versus NRT on smoking cessation was determined using three statistical approaches to try to overcome confounding. Firstly, we fitted multivariable logistic regression models adjusting for all covariates and using robust standard errors to account for potential clustering within practices. These analyses were conducted in the following patient groups: no neurodevelopmental disorder, any neurodevelopmental disorder, ASD, ADHD, and intellectual disabilities. Secondly, we repeated the analyses using propensity score matched logistic regression. Developing the propensity score model involved fitting a logistic regression model including all baseline covariates to calculate each participant’s propensity score. Then, within each neurodevelopmental disorder group, each person prescribed varenicline was matched to another person prescribed NRT with the closest propensity score in a ratio of 1:1 using a nearest neighbour algorithm with no replacement, and matching was restricted to the common support region^40^. Those who could not be matched were discarded from that analysis. Thirdly, we utilized instrumental variable regression analyses, with physicians’ previously recorded prescribing preferences for varenicline versus NRT as the instrument. The instrument (physicians’ prescribing preference) was defined by the seven smoking cessation prescriptions previously issued by the physician to their previous patients before their current patient. Physicians who previously prescribed varenicline were categorised as a varenicline prescriber^41^. The instrumental variable regression models were developed in two groups (patients with and without neurodevelopmental disorders) due to insufficient power to display meaningful results by individual neurodevelopmental disorders. Instrumental variable assumptions for our study were: (1) the physicians’ prescribing preference was associated with the probability they will prescribe varenicline rather than NRT to their patient; (2) the physicians’ prescribing preference for varenicline or NRT does not affect smoking cessation other than through the decision on whether to prescribe varenicline or NRT; and (3) the physicians’ prescribing preference for varenicline or NRT is not related to characteristics of their patient population. The instrument used in this study was previously reported as a potential approach to control for confounding by indication in nonexperimental studies^16,20,41,42^. All analyses were conducted using Stata 14 MP.

#### Missing data

Patients with missing smoking data during the follow-up period (beyond 180 days) were assumed to be continuing smokers^43^. This has been shown to be robust to sensitivity analyses^16^. We used multiple imputation to handle missing data on BMI and IMD. This was done using the ICE command in Stata where we produced 20 imputed datasets (eTable 2). We included all exposures, covariates, and outcomes in the imputation model^26^.

### Ethical considerations

The study was approved by the Independent Scientific Advisory Committee (ISAC) on 04/06/2015 (protocol number 15_115). Patient and practice confidentiality was maintained in accordance with the CPRD policy on personal data (https://www.cprd.com/dataAccess/).

### Data statement

This study is based in part on data from the Clinical Practice Research Datalink obtained under licence from the UK Medicines and Healthcare products Regulatory Agency. The data are provided by patients and collected by the NHS as part of their care and support. The interpretation and conclusions contained in this study are those of the authors alone.

### Data access

The data can be accessed by submitting an application to ISAC: https://cprd.com/Data-access. Codelists that were used for this study are available upon request from the corresponding author.

## Supplementary information

Supplementary information
