# Supplementary material for: Long-term effectiveness and safety of varenicline and nicotine replacement therapy in people with neurodevelopmental disorders: A prospective cohort study
Source: Sci Rep. 2019 Dec 20;9:19488. doi: 10.1038/s41598-019-54727-5 (PMC6925148; doi:10.1038/s41598-019-54727-5)
Supplement: Supplementary file 1 — Supplementary information [file 41598_2019_54727_MOESM1_ESM.docx]

## Supplementary Material

Long-term effectiveness and safety of varenicline and nicotine replacement therapy in people with neurodevelopmental disorders: A prospective cohort study

**Authors**

Taha Itani PhD^1,2^; Dheeraj Rai PhD^3,4,5^, Tim Jones PhD^6^; Gemma MJ Taylor PhD^7^; Kyla H Thomas PhD^8^; Richard M Martin PhD^1,5, 9^; Marcus R Munafò PhD^1,2^; Neil M Davies PhD^1,9^ and Amy E Taylor PhD^5,9^

**Author affiliations**

1 Medical Research Council Integrative Epidemiology Unit at the University of Bristol, BS8 2BN, United Kingdom.

2 UK Centre for Tobacco and Alcohol Studies, School of Psychological Science, University of Bristol, 12a Priory Road, Bristol, BS8 1TU, United Kingdom.

3 Centre for Academic Mental Health, Bristol Medical School, University of Bristol, Barley House, Oakfield Grove, Bristol, BS8 2BN, United Kingdom.

4 Avon & Wiltshire Partnership NHS Mental Health Trust, Bristol, UK

5 NIHR Biomedical Research Centre at the University Hospitals Bristol NHS Foundation Trust and the University of Bristol, United Kingdom.

6 National Institute for Health Research Collaboration for Leadership in Applied Health Research and Care West (NIHR CLAHRC West) at University Hospitals Bristol NHS Foundation Trust, 9^th^ Floor Whitefriars, Lewins Mead, Bristol, BS1 2NT, United Kingdom.

7Addiction and Mental Health Group (AIM) Department of Psychology, University of Bath, Claverton Down, Bath, BA2 7AY.

8 Population Health Sciences, Bristol Medical School, University of Bristol, Canynge Hall, Bristol, BS8 2PS, United Kingdom.

## eFigure 1. Flow chart of eligible study participants as per protocol restrictions

| Excluded from analysis due to protocol restrictions (Davies et al. 2015) | | |
| --- | --- | --- |
|  | Prescriptions | Patients |
| Prescriptions issued to patients under the age of 15 | 6580 | 1944 |
| Prescription issued after patients’ registration period ended | 0 | 0 |
| Prescription issued before patients’ registration period began | 296490 | 53529 |
| Prescription issued to patient aged 16 or 17 | 13154 | 4425 |
| Prescription issued before 1st September 2006 | 822244 | 145382 |
| Both varenicline and NRT were prescribed on the same day | 8289 | 264 |
| Smoking cessation medication was not prescribed by a general practitioner (GP) | 141810 | 16168 |
| Smoking cessation medication prescription had less than one year of historical follow-up data prior to prescription | 163821 | 41112 |
| Bupropion prescriptions | 32997 | 6981 |
| Prescriptions issued to patients who received a smoking cessation medication issued in the previous 18 months | 1069188 | 30278 |
| Patient had previously received an eligible smoking cessation medication prescription | 55870 | 0 |
| Prescribing GP had seen less than 10 patients. | 18494 | 18494 |
| Patients had less than 180 days of follow-up after 1^st^ prescription. | 13045 | 13045 |
|  |  |  |
| Total excluded N= | 2,641,982 | 331,622 |

Assessed for eligibility

N Records=2,877,296

N Patients=566,936

Varenicline

N patients=75,578

NRT

N patients=159,736

Included in analysis

N patients=235,314

## eFigure 2. Age and sex standardised percentage (%) of primary care patients with an electronic medical record indicating smoking, from 2004 to 2015, stratified by people with neurodevelopmental disorders

eTable 1. Raw numbers used for generating the prevalence rates from 2004 to 2015, stratified by people with and without neurodevelopmental disorders

| **Autism** | | | **ADHD** | | **Intellectual disabilities** | | **Any neurodevelopmental disorder** | | **No neurodevelopmental disorder** | |
| --- | --- | --- | --- | --- | --- | --- | --- | --- | --- | --- |
| **Year** | N smokers | N Autism | N smokers | N ADHD | N smokers | N Intellectual disabilities | N smokers | N any neurodevelopmental disorders | N smokers | N no neurodevelopmental disorders |
| **2004** | 404 | 2,203 | 1,537 | 3,242 | 5,037 | 21,701 | 6,685 | 25,583 | 890,340 | 3,243,475 |
| **2005** | 493 | 2,649 | 1,923 | 3,953 | 5,665 | 23,995 | 7,707 | 28,670 | 951,394 | 3,502,017 |
| **2006** | 595 | 3,110 | 2,428 | 4,869 | 6,195 | 26,024 | 8,762 | 31,707 | 985,018 | 3,700,096 |
| **2007** | 716 | 3,672 | 2,944 | 5,872 | 6,829 | 28,120 | 9,909 | 34,881 | 1,003,586 | 3,852,720 |
| **2008** | 836 | 4,232 | 3,543 | 6,933 | 7,278 | 29,395 | 10,935 | 37,317 | 1,006,176 | 3,887,889 |
| **2009** | 993 | 4,842 | 4,198 | 8,197 | 7,787 | 30,781 | 12,078 | 40,039 | 1,009,313 | 3,928,689 |
| **2010** | 1,142 | 5,504 | 4,803 | 9,353 | 8,214 | 32,101 | 13,073 | 42,668 | 1,005,960 | 3,946,830 |
| **2011** | 1,282 | 6,210 | 5,358 | 10,364 | 8,389 | 32,730 | 13,837 | 44,521 | 977,034 | 3,883,994 |
| **2012** | 1,406 | 6,862 | 5,842 | 11,398 | 8,306 | 32,901 | 14,253 | 45,906 | 920,043 | 3,822,908 |
| **2013** | 1,526 | 7,638 | 6,208 | 12,377 | 7,930 | 33,031 | 14,225 | 47,226 | 855,420 | 3,788,298 |
| **2014** | 1,474 | 7,763 | 6,037 | 12,280 | 7,196 | 31,095 | 13,315 | 45,347 | 737,802 | 3,531,275 |
| **2015** | 1,387 | 7,441 | 5,522 | 11,544 | 6,230 | 27,416 | 11,832 | 40,946 | 619,302 | 3,064,046 |

## eTable 2. Distributions of imputed characteristics in the imputation datasets

| **Characteristic** | **Any neurodevelopmental disorder**  **(N=2,346)** | | | | | | | **No neurodevelopmental disorder**  **(N=232,968)** | | | | | | |
| --- | --- | --- | --- | --- | --- | --- | --- | --- | --- | --- | --- | --- | --- | --- |
|  | **NRT (N=1,882)** | | **Varenicline (N=464)** | | **Total** | | **% of data imputed** | **NRT (N=157,854)** | | **Varenicline (N=75,114)** | | **Total** | | **% of data imputed** |
|  | Mean | Standard error | Mean | Standard error | Mean | Standard error |  | Mean | Standard error | Mean | Standard error | Mean | Standard error |  |
| Body mass index | 26.9 | 0.16 | 26.2 | 0.31 | 26.7 | 0.15 | 18.5 | 26.4 | 0.02 | 26.5 | 0.02 | 26.4 | 0.01 | 14.0 |
| Index of multiple deprivation | 3.53 | 0.04 | 3.42 | 0.07 | 3.51 | 0.03 | 43.0 | 3.30 | 0.004 | 3.18 | 0.007 | 3.26 | 0.004 | 43.6 |

## eTable 3. The likelihood of smokers with neurodevelopmental disorders being prescribed varenicline versus NRT, as compared to smokers with no neurodevelopmental disorder, N=235,314

|  | **Partially adjusted odds ratio**  **(95% confidence interval) †** | **Fully adjusted odds ratio**  **(95% confidence interval) ††** |
| --- | --- | --- |
| Any neurodevelopmental disorder | 0.41 (0.37 to 0.46) | 0.52 (0.46 to 0.58) |
| Autism | 0.23 (0.16 to 0.33) | 0.30 (0.21 to 0.43) |
| ADHD | 0.45 (0.37 to 0.55) | 0.51 (0.42 to 0.64) |
| Intellectual disabilities | 0.41 (0.36 to 0.47) | 0.53 (0.46 to 0.61) |
| ‡Partially adjusted models were adjusted for: age, sex, year of first prescription.  †† Fully adjusted models were adjusted for: age, sex, days in history, IMD, number of GP visits 1-year prior to first prescription, BMI, year of first prescription, history of major physical morbidity (Charlson Index), alcohol misuse ever, drug misuse ever, depression ever, neurotic disorder ever, self-harm ever, schizophrenia ever, antidepressant prescription ever, antipsychotic prescription ever, hypnotics/anxiolytics prescription ever, other psychotropic medication ever, and other behavioral/neurologic disorder ever. Missing BMI and IMD values were imputed using multiple imputation.  Models were estimated using cluster robust standard errors to account for potential clustering of patients between practices. | | |

eTable 3 presents Partially and fully adjusted odds ratios and 95% confidence intervals for likelihood of those with neurodevelopmental disorders being prescribed varenicline versus NRT, as compared to smokers with no neurodevelopmental disorders. Smokers with neurodevelopmental disorders were less likely to be prescribed varenicline compared to smokers with no neurodevelopmental disorders.

## eTable 4. Number and percentage (%) of patients with an electronic medical record indicating smoking cessation at 3, 6 and 9-months, and 1, 2, and 4-years follow-up by exposure group, and by neurodevelopmental disorder

|  | **Treatment** | **3-months** | **6-months** | **9-months** | **1-year** | **2-years** | **4-years** |
| --- | --- | --- | --- | --- | --- | --- | --- |
| No neurodevelopmental disorder | NRT (N=157,854) | 22034 (14.0%) | 27950 (17.7%) | 31052 (19.7%) | 33316 (21.1%) | 38316 (24.3%) | 45133 (28.6%) |
|  | Varenicline (N=75,114) | 13994 (18.6%) | 17588 (23.4%) | 18851 (25.1%) | 19652 (26.2%) | 21583 (28.7%) | 24375 (32.5%) |
| Any neurodevelopmental disorder | NRT (N=1,882) | 182 (9.7%) | 227 (12.1%) | 259 (13.8%) | 292 (15.5%) | 333 (17.7%) | 364 (19.3%) |
|  | Varenicline (N=464) | 60 (12.9%) | 82 (17.7%) | 92 (19.8%) | 90 (19.4%) | 104 (22.4%) | 110 (23.7%) |
| Autism | NRT (N=223) | 16 (7.2%) | 18 (8.0%) | 24 (10.8%) | 28 (12.6%) | 31 (13.9%) | 42 (18.8%) |
|  | Varenicline (N=35) | 8 (22.9%) | 9 (25.7%) | 9 (25.7%) | 9 (25.7%) | 9 (25.7%) | 9 (25.7%) |
| ADHD | NRT (N=405) | 28 (6.9%) | 35 (8.6%) | 42 (10.4%) | 55 (13.6%) | 67 (16.5%) | 83 (20.5%) |
|  | Varenicline (N=119) | 13 (10.9%) | 16 (13.4%) | 22 (18.5%) | 18 (15.1%) | 24 (20.2%) | 30 (25.2%) |
| Intellectual disabilities | NRT (N=1,394) | 144 (10.3%) | 184 (13.2%) | 205 (14.7%) | 225 (16.1%) | 254 (18.2%) | 261 (18.7%) |
|  | Varenicline (N=328) | 42 (12.8%) | 61 (18.6%) | 65 (19.8%) | 68 (20.7%) | 75 (22.9%) | 75 (22.9%) |

## eTable 5. Stratified by neurodevelopmental disorder: Partially adjusted odds ratios and 95% confidence intervals for the association between prescription of varenicline versus NRT and smoking cessation at 3, 6 and 9-months and 1, 2, and 4-years after prescription

| **Partially adjusted odds ratio (95% confidence interval) †** | | | | | | |
| --- | --- | --- | --- | --- | --- | --- |
|  | 3-months | 6-months | 9-months | 1-year | 2-years | 4-years |
| No neurodevelopmental disorder (N=232,968) | 1.47  (1.42 to 1.52) | 1.50  (1.46 to 1.54) | 1.44  (1.40 to 1.48) | 1.38  (1.34 to 1.42) | 1.29  (1.26 to 1.32) | 1.22  (1.20 to 1.25) |
| Any neurodevelopmental disorder (N=2,344) | 1.47  (1.06 to 2.03) | 1.68  (1.28 to 2.21) | 1.64  (1.26 to 2.13) | 1.35  (1.03 to 1.76) | 1.40  (1.09 to 1.80) | 1.35  (1.05 to 1.73) |
| Autism (N=258) | 3.77  (1.31 to 10.87) | 3.63  (1.34 to 9.84) | 2.68  (1.03 to 6.98) | 2.13  (0.82 to 5.55) | 1.90  (0.75 to 4.81) | 1.26  (0.51 to 3.07) |
| ADHD (N=524) | 1.59  (0.77 to 3.29) | 1.58  (0.81 to 3.08) | 1.96  (1.10 to 3.49) | 1.09  (0.61 to 1.94) | 1.21  (0.71 to 2.07) | 1.26  (0.77 to 2.08) |
| Intellectual disability (N=1,720) | 1.42  (0.96 to 2.12) | 1.66  (1.20 to 2.29) | 1.55  (1.13 to 2.13) | 1.42  (1.04 to 1.95) | 1.39  (1.03 to 1.87) | 1.34  (0.99 to 1.81) |
| ‡ Partially adjusted models were adjusted for: age, sex, year of first prescription. Models were estimated using cluster robust standard errors to account for potential clustering of patients between practices. | | | | | | |

## eTable 6. Stratified by neurodevelopmental disorder: Propensity score matched logistic regression odds ratios and 95% confidence intervals for the association between prescription of varenicline versus NRT and smoking cessation at 3, 6 and 9-months, and 1, 2, and 4-years follow-up

| **Propensity score matched logistic regression**  **Adjusted odds ratio (95% confidence interval) *** | | | | | | |
| --- | --- | --- | --- | --- | --- | --- |
|  | 3-months | 6-months | 9-months | 1-year | 2-years | 4-years |
| No neurodevelopmental disorder (N=150,224) | 1.41  (1.36 to 1.47) | 1.43  (1.39 to 1.48) | 1.38  (1.33 to 1.42) | 1.32  (1.29 to 1.37) | 1.24  (1.20 to 1.27) | 1.18  (1.15 to 1.21) |
| Any neurodevelopmental disorder (N=928) | 1.39  (0.87 to 2.23) | 1.61  (1.04 to 2.51) | 1.63  (1.07 to 2.46) | 1.33  (0.88 to 2.01) | 1.37  (0.94 to 1.98) | 1.32  (0.93 to 1.88) |
| Autism (N=66) | 4.28  (0.65 to 28.14) | 3.45  (0.69 to 17.30) | 3.08  (0.62 to 15.23) | 3.06  (0.51 to 18.52) | 2.53  (0.56 to 11.52) | 1.94  (0.42 to 8.98) |
| ADHD (N=234) | 1.54  (0.56 to 4.22) | 1.39  (0.57 to 3.39) | 1.88  (0.79 to 4.48) | 1.03  (0.46 to 2.30) | 1.20  (0.59 to 2.46) | 1.29  (0.64 to 2.58) |
| Intellectual disability (N=654) | 1.38  (0.76 to 2.50) | 1.59  (0.97 to 2.59) | 1.54  (0.94 to 2.55) | 1.48  (0.91 to 2.43) | 1.41  (0.89 to 2.25) | 1.35  (0.83 to 2.18) |
| *****Estimates were adjusted for propensity score. Models were estimated using cluster robust standard errors to account for potential clustering of patients between practices. | | | | | | |

## eTable 7. Stratified by neurodevelopmental disorder: Instrumental variable regression risk difference per 100 patients and 95% confidence intervals for the association between prescription of varenicline versus NRT and smoking cessation at 3, 6 and 9-months, and 1, 2, and 4-years follow-up

| **Linear regression**  **Risk difference per 100 patients (95% confidence interval) ‡‡** | | | | | | |
| --- | --- | --- | --- | --- | --- | --- |
|  | **3-months** | **6-months** | **9-months** | **1-year** | **2-years** | **4-years** |
| No neurodevelopmental disorder (N=203,131) | 4.27  (2.50 to 6.05) | 6.60  (4.75 to 8.46) | 6.78  (4.95 to 8.60) | 6.63  (4.83 to 8.42) | 5.31  (3.67 to 6.95) | 4.56  (2.99 to 6.12) |
| Any neurodevelopmental disorder (N=2,101) * | 8.24  (-1.11 to 17.59) | 10.96  (0.90 to 21.03) | 13.08  (2.34 to 23.82) | 6.11  (-4.88 to 17.11) | 11.98  (0.29 to 23.67) | 11.57  (-0.42 to 23.57) |
| ‡‡Partially adjusted estimates were adjusted for year of prescription. * Models were estimated using cluster robust standard errors to account for potential clustering of patients between practices. *Missing instrumental variable data for patients who were the first patient to be prescribed either varenicline or NRT by their GP during the study period. | | | | | | |

## eTable 8. Partially adjusted odds ratios and 95% confidence intervals for the association between prescription of varenicline versus NRT and self-harm, depression, antidepressants, anxiety/stress related disorders, and hypnotics/anxiolytics at 3, 6 and 9-months and 1, 2, and 4-years after prescription amongst people with any neurodevelopmental disorders (N=2,346)

| **Partially adjusted odds ratio (95% confidence interval) †** | | | | | | |
| --- | --- | --- | --- | --- | --- | --- |
|  | 3-months | 6-months | 9-months | 1-year | 2-years | 4-years |
| Self-harm | 0.58  (0.07 to 4.65) | 0.86  (0.19 to 3.97) | 0.88  (0.24 to 3.17) | 1.17  (0.42 to 3.25) | 1.0  (0.42 to 2.34) | 0.85  (0.41 to 1.78) |
| Depression | 0.28  (0.04 to 2.12) | 0.72  (0.28 to 1.82) | 1.23  (0.64 to 2.36) | 1.55  (0.89 to 2.69) | 1.33  (0.83 to 2.13) | 1.05  (0.70 to 1.58) |
| Antidepressants | 0.39  (0.29 to 0.52) | 0.46  (0.36 to 0.60) | 0.52  (0.41 to 0.67) | 0.55  (0.43 to 0.69) | 0.62  (0.50 to 0.77) | 0.65  (0.53 to 0.81) |
| Anxiety and stress related disorders | 1.19  (0.30 to 4.67) | 1.81  (0.69 to 4.74) | 1.67  (0.76 to 3.68) | 1.35  (0.71 to 2.55) | 1.29  (0.77 to 2.15) | 1.12  (0.74 to 1.71) |
| Hypnotics/anxiolytics | 0.37  (0.24 to 0.56) | 0.47  (0.33 to 0.67) | 0.57  (0.42 to 0.78) | 0.64  (0.48 to 0.86) | 0.65  (0.50 to 0.84) | 0.65  (0.51 to 0.83) |
| ‡ Partially adjusted models were adjusted for: age, sex, year of first prescription. Models were estimated using cluster robust standard errors to account for potential clustering of patients between practices. | | | | | | |

## eTable 9. Fully adjusted odds ratios and 95% confidence intervals for the association between prescription of varenicline versus NRT and self-harm, depression, antidepressants, anxiety/stress related disorders, and hypnotics/anxiolytics at 3, 6 and 9-months and 1, 2, and 4-years after prescription amongst people with any neurodevelopmental disorders (N=2,346)

| **Fully adjusted odds ratio (95% confidence interval) †** | | | | | | |
| --- | --- | --- | --- | --- | --- | --- |
|  | 3-months | 6-months | 9-months | 1-year | 2-years | 4-years |
| Self-harm | 0.60  (0.06 to 6.04) | 0.78  (0.15 to 3.97) | 0.78  (0.19 to 3.13) | 1.04  (0.33 to 3.26) | 0.90  (0.36 to 2.28) | 0.89  (0.42 to 1.92) |
| Depression | 0.33  (0.04 to 2.57) | 0.81  (0.29 to 2.21) | 1.23  (0.62 to 2.46) | 1.53  (0.85 to 2.74) | 1.35  (0.81 to 2.23) | 0.98  (0.63 to 1.53) |
| Antidepressants | 0.42  (0.30 to 0.59) | 0.52  (0.38 to 0.73) | 0.61  (0.45 to 0.83) | 0.64  (0.48 to 0.85) | 0.75  (0.57 to 0.97) | 0.78  (0.61 to 1.01) |
| Anxiety and stress related disorders | 0.96  (0.22 to 4.16) | 1.73  (0.61 to 4.88) | 1.67  (0.72 to 3.86) | 1.26  (0.63 to 2.50) | 1.19  (0.70 to 2.04) | 1.09  (0.70 to 1.69) |
| Hypnotics/anxiolytics | 0.43  (0.27 to 0.68) | 0.58  (0.39 to 0.86) | 0.73  (0.51 to 1.03) | 0.83  (0.60 to 1.14) | 0.83  (0.61 to 1.12) | 0.82  (0.63 to 1.08) |
| **†** Fully adjusted models were adjusted for: age, sex, days in history, IMD, number of GP visits 1-year prior to first prescription, BMI, year of first prescription, history of major physical morbidity (Charlson Index), alcohol misuse ever, drug misuse ever, depression ever, neurotic disorder ever, self-harm ever, schizophrenia ever, antidepressant prescription ever, antipsychotic prescription ever, hypnotics/anxiolytics prescription ever, other psychotropic medication ever, and other behavioral/neurologic disorder ever. Missing BMI and IMD values were imputed using multiple imputation. | | | | | | |

## eTable 10. Propensity score matched logistic regression odds ratios and 95% confidence intervals for the association between prescription of varenicline versus NRT and self-harm, depression, antidepressants, anxiety/stress related disorders, and hypnotics/anxiolytics at 3, 6 and 9-months and 1, 2, and 4-years after prescription amongst people with any neurodevelopmental disorders (N=928)

| **Propensity score matched logistic regression**  **Adjusted odds ratio (95% confidence interval) *** | | | | | | |
| --- | --- | --- | --- | --- | --- | --- |
|  | 3-months | 6-months | 9-months | 1-year | 2-years | 4-years |
| Self-harm | 0.55  (0.03 to 8.03) | 0.86  (0.11 to 7.02) | 0.84  (0.14 to 5.06) | 1.12  (0.27 to 4.66) | 0.99  (0.28 to 3.54) | 0.82  (0.29 to 2.26) |
| Depression | 0.34  (0.03 to 3.95) | 0.77  (0.22 to 2.77) | 1.14  (0.42 to 3.07) | 1.42  (0.61 to 3.30) | 1.24  (0.63 to 2.45) | 0.97  (0.55 to 1.72) |
| Antidepressants | 0.51  (0.35 to 0.76) | 0.61  (0.43 to 0.87) | 0.67  (0.48 to 0.94) | 0.68  (0.50 to 0.94) | 0.77  (0.56 to 1.06) | 0.78  (0.57 to 1.08) |
| Anxiety and stress related disorders | 0.88  (0.15 to 5.10) | 1.47  (0.35 to 6.07) | 1.38  (0.46 to 4.20) | 1.23  (0.45 to 2.81) | 1.12  (0.53 to 2.37) | 1.01  (0.54 to 1.88) |
| Hypnotics/anxiolytics | 0.52  (0.29 to 0.91) | 0.65  (0.41 to 1.04) | 0.79  (0.52 to 1.20) | 0.84  (0.57 to 1.25) | 0.88  (0.61 to 1.28) | 0.87  (0.63 to 1.22) |
| *****Estimates were adjusted for propensity score. Models were estimated using cluster robust standard errors to account for potential clustering of patients between practices. | | | | | | |

## eTable 11. Instrumental variable regression risk difference per 100 patients and 95% confidence intervals for the association between prescription of varenicline versus NRT and self-harm, depression, antidepressants, anxiety/stress related disorders, and hypnotics/anxiolytics at 3, 6 and 9-months, and 1, 2, and 4-years after prescription amongst people with any neurodevelopmental disorders (N=2,101) *

| **Linear regression**  **Risk difference per 100 patients (95% confidence interval) ‡‡** | | | | | | |
| --- | --- | --- | --- | --- | --- | --- |
|  | **3-months** | **6-months** | **9-months** | **1-year** | **2-years** | **4-years** |
| Self-harm | -0.32  (-1.20 to 0.56) | 0.38  (-1.47 to 2.22) | 0.59  (-1.70 to 2.88) | 0.74  (-1.71 to 3.19) | -0.62  (-3.60 to 2.36) | -2.45  (-6.21 to 1.30) |
| Depression | -1.81  (-3.61 to -0.01) | -0.97  (-3.79 to 1.85) | 0.78  (-3.44 to 5.01) | 2.08  (-2.50 to 6.66) | 2.89  (-2.48 to 8.26) | 0.66  (-5.74 to 7.06) |
| Antidepressants | -14.44  (-26.39 to -2.49) | -12.59  (-25.70 to 0.51) | -13.59  (-27.54 to 0.37) | -11.34  (-25.39 to 2.71) | -9.14  (-24.41 to 6.13) | -10.93  (-25.95 to 4.08) |
| Anxiety and stress related disorders | 0.56  (-2.28 to 3.40) | 0.05  (-3.25 to 3.36) | 0.51  (-3.71 to 4.72) | 0.69  (-3.79 to 5.18) | 1.13  (-4.53 to 6.80) | -3.10  (-9.74 to 3.54) |
| Hypnotics/anxiolytics | -3.30  (-12.49 to 5.89) | -3.81  (-14.39 to 6.77) | -4.19  (-15.34 to 6.95) | -7.33  (-19.18 to 4.52) | -14.94  (-28.08 to -1.81) | -17.43  (-31.17 to -3.69) |
| ‡‡Partially adjusted estimates were adjusted for year of prescription. Models were estimated using cluster robust standard errors to account for potential clustering of patients between practices.  *Missing instrumental variable data for patients who were the first patient to be prescribed either varenicline or NRT by their GP during the study period. | | | | | | |
